# EAGA-MLP—An Enhanced and Adaptive Hybrid Classification Model for Diabetes Diagnosis

**DOI:** 10.3390/s20144036

**Published:** 2020-07-20

**Authors:** Sushruta Mishra, Hrudaya Kumar Tripathy, Pradeep Kumar Mallick, Akash Kumar Bhoi, Paolo Barsocchi

**Affiliations:** 1 School of Computer Engineering, Kalinga Institute of Industrial Technology, Deemed to be University, Bhubaneswar 751024, India; hktripathyfcs@kiit.ac.in (H.K.T.); pradeep.mallickfcs@kiit.ac.in (P.K.M.); 2 Department of Electrical and Electronics Engineering, Sikkim Manipal Institute of Technology, Sikkim Manipal University, Majhitar, Sikkim 737136, India; akash.b@smit.smu.edu.in; 3 Institute of Information Science and Technologies, National Research Council, 56124 Pisa, Italy

**Keywords:** diabetes, classification, attribute optimization, genetic algorithm, classification accuracy, F-Score, mutation, fitness function

## Abstract

Disease diagnosis is a critical task which needs to be done with extreme precision. In recent times, medical data mining is gaining popularity in complex healthcare problems based disease datasets. Unstructured healthcare data constitutes irrelevant information which can affect the prediction ability of classifiers. Therefore, an effective attribute optimization technique must be used to eliminate the less relevant data and optimize the dataset for enhanced accuracy. Type 2 Diabetes, also called Pima Indian Diabetes, affects millions of people around the world. Optimization techniques can be applied to generate a reliable dataset constituting of symptoms that can be useful for more accurate diagnosis of diabetes. This study presents the implementation of a new hybrid attribute optimization algorithm called Enhanced and Adaptive Genetic Algorithm (EAGA) to get an optimized symptoms dataset. Based on readings of symptoms in the optimized dataset obtained, a possible occurrence of diabetes is forecasted. EAGA model is further used with Multilayer Perceptron (MLP) to determine the presence or absence of type 2 diabetes in patients based on the symptoms detected. The proposed classification approach was named as Enhanced and Adaptive-Genetic Algorithm-Multilayer Perceptron (EAGA-MLP). It is also implemented on seven different disease datasets to assess its impact and effectiveness. Performance of the proposed model was validated against some vital performance metrics. The results show a maximum accuracy rate of 97.76% and 1.12 s of execution time. Furthermore, the proposed model presents an F-Score value of 86.8% and a precision of 80.2%. The method is compared with many existing studies and it was observed that the classification accuracy of the proposed Enhanced and Adaptive-Genetic Algorithm-Multilayer Perceptron (EAGA-MLP) model clearly outperformed all other previous classification models. Its performance was also tested with seven other disease datasets. The mean accuracy, precision, recall and f-score obtained was 94.7%, 91%, 89.8% and 90.4%, respectively. Thus, the proposed model can assist medical experts in accurately determining risk factors of type 2 diabetes and thereby help in accurately classifying the presence of type 2 diabetes in patients. Consequently, it can be used to support healthcare experts in the diagnosis of patients affected by diabetes.

## 1. Introduction

Type 1 and type 2 are two primary categories of diabetes which are affecting people throughout world. Both these types are chronic. Persons affected with type 1 diabetes are unable to generate insulin while individuals detected with type 2 diabetes fail to respond to insulin and in the long run cannot produce insulin. Pima Indian Diabetes is a potentially life threatening disease that may create serious worldwide havoc. Type 2 Diabetes is also referred to as Pima Indian Diabetes. It can have serious complications on our heart, kidney and eyes. As per the International Diabetes confederation, 382 million people are affected with this disease worldwide. By 2035, this figure may get doubled to 592 million [1]. Determining factors and symptoms of diabetes during initial phase is of the utmost importance. Diabetes treatment concentrates on controlling glucose levels to avert different side effects and entanglements through the solution, eating regimen and exercise. This disease, if not treated legitimately and on timely basis, can create intense entanglements and even loss of life [2,3]. Due to the rapid rise in diabetic cases and increased complexity in massive data records of diabetic patients, it is becoming increasingly difficult for medical experts to provide effective treatment manually [4]. Hence for better diagnosis of a diabetic patient, medical data mining can be successfully implemented since it enables the detection of diabetes in an earlier stage. Data Classification technique can be used to categorize diabetes patients from non-diabetic ones. Nevertheless, certain irrelevant and ambiguous Attributes exist in raw unstructured disease datasets. Due to the presence of such Attributes, the overall efficiency of classification in data mining is affected. Consequently, an effective attribute optimization method can be used to eliminate these less relevant data and generate an optimized dataset with vital symptoms which can be accurately mined using a suitable classification algorithm [3]. The attribute optimization method acts as an optimizing agent, which can be successfully applied to massive and complex datasets to reduce the sample size without compromising any critical data. Therefore, attribute optimization minimizes the execution time and improve the effectiveness of classification.

The treatment of diseases starts with the proper identification of the symptoms. To deal with a widespread disease like diabetes, the detection of its symptoms on time is essential. In a disease like diabetes, various risk factors are involved and moreover data instances consist of missing values, redundant values and other inconsistencies. As a result, sometimes even with lesser data samples detecting important symptoms become difficult. If correct symptoms are not selected, then it affects the performance in prediction and classification. Overall the disease diagnosis is affected. In a diabetic patient, some common symptoms include increased thirst, frequent urination, increased hunger, unintended weight loss, fatigue, blurred vision and slow healing sores. There are presence of missing values and duplicate values in the dataset under consideration, which needs to be removed. An attribute selector can be used to handle this issue. The core purpose of our study is to discuss a new attribute optimization model which is based on genetic algorithm to help in precise identification of relevant symptoms which can be used to predict the likelihood of a person getting affected with diabetes in future. Based on the recorded readings of symptoms, a person can be notified and alerted about a possible occurrence of diabetes in future. Then on basis of the relevant symptoms obtained, effective classification can be carried out to determine the presence of diabetes. Thus, it provides an accurate and fast diagnosis of type 2 diabetes. Hence the primary contribution of this study is to present the development and implementation of an Enhanced and Adaptive Genetic Algorithm (EAGA) to be used on diabetes dataset and then using Multi-layer Perceptron (MLP) model to classify between diabetes and non-diabetes patients. The purpose of the work is to make early diagnosis of type 2 diabetes based on symptoms observed in patients seeking medical help. Based on symptoms seen in patients, our proposed classification model can assist medical experts to differentiate diabetic from non-diabetic patients so that appropriate medical attention is provided to them at an early stage.

**Figure 1:**
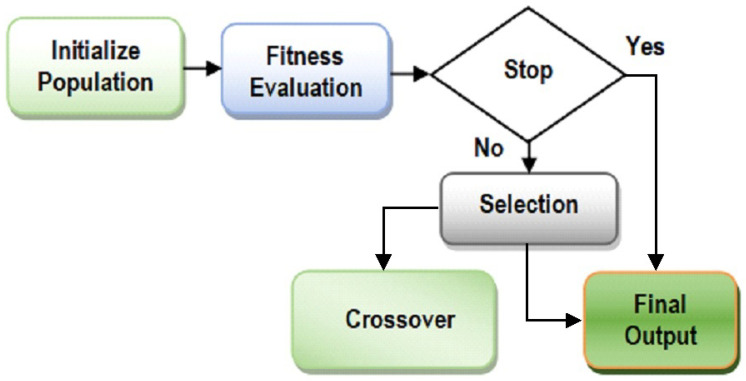
Overview of Genetic Algorithm.

The Pima Indian Diabetes (http://networkrepository.com/pima-indians-diabetes.php) dataset is used in this work. Our analysis provides a positive impact of attribute optimization on the diabetes dataset, which can assist the healthcare professionals to determine the presence of diabetes in patients on the basis of their symptoms. The presented attribute optimization technique is an adaptation of the genetic algorithm method. A different initial solution space is taken with a new and improved adaptation of crossover and mutation phase in a genetic algorithm. A new variation of the fitness function is developed and used in the study. The new enhanced attribute optimization method is named Enhanced and Adaptive Genetic Algorithm (EAGA). The Genetic Algorithm is related to the idea of “survival of the fittest” and it imitates the process of Natural Selection. These algorithms are effective in determining solutions related to search and optimization problems. It operates on three basic principles of nature, which include selection, crossover and mutation. These operations are run in a loop until specific conditions are satisfied. The steps of GA are highlighted in Figure 1.

This algorithm generates an optimized diabetes dataset which is further partitioned into 60% training set and 40% testing set. The MLP (Multi Layer Perceptron) is the classifier used in our research study. The classification model is first trained and then it is evaluated with the testing set using specific performance metrics like accuracy rate, precision and recall.

This paper is arranged into different distinct sections. The Section 2 presents a literature survey where several vital existing works related to diabetes prediction and analysis using various algorithms and models are highlighted. The Section 3 is the core part of the study. It presents the methodology, where the Pima Indian diabetes dataset details are provided and the proposed technique of attribute optimization and classification is outlined with a diagrammatic explanation. Several pseudo-codes of the different steps are presented. Then the Section 4 illustrates an experimental demonstration of our proposed model with simulation results. The Section 5 analyses the results obtained in work along with its inferences. Finally, the paper is concluded with the main findings and analysis of implementation in the Section 6.

## 2. Literature Review

Several research works are being carried out by different authors for the effective treatment of diabetes using machine learning. In this section, relevant similar works on diabetes analysis and prediction are discussed.

In Reference [4], a proposal was presented to uncover the hidden patterns to enhance the health facilities for patients who have diabetes. It gave an insight into frame hidden variables that can predict the intensity of diabetes mellitus symptoms on patients. Patient records and clinical tests were analyzed to uncover hidden trends in diabetes datasets to improve the quality of living of diabetic patients. In this analysis, decision tree and rule based classifiers were mainly used for classification.

Ref. [5] predicted the patterns of diabetes on people with distinct age groups and lifestyles. The study discussed ways to deal with missing values in diabetes dataset. Neural network was used for classification and when it was used with some pre-processing methods, it produced an optimum accuracy of 99%.

A dimensional reduction in heterogeneous diabetes dataset using Self Organizing Map (SOM) clustering was performed and established similarities among patients using (Unified distance) U-Matrix [6]. Questionnaires consisting of both text as well as numeric responses were used. SOM was used as a data visualization tool. It aimed at interpreting patient’s behavior and interlinking diabetes factors with each other to show the correlation among them. The output was illustrated in U-matrix format. Researchers in Reference [7] developed a classification and risk analysis framework for diabetes and hypertension on clinical centers in Kuwait. The authors compared the performance of four different classifiers, including multifactor dimensionality reduction, logistic regression, k-nearest neighbors and Support Vector Machines (SVM) using non-laboratory attributes. It gave an accuracy rate of 85% for diabetes and more than 90% for hypertension disorder. Classification with K-NN algorithm gave the highest risk of 75% in diabetic patients and 94% in hypertension patients.

The decision tree model for predicting symptoms in diabetic patients is discussed in Reference [8]. The model comprised two phases which include data pre-processing and data prediction. In data pre-processing phase, relevant attributes were selected and missing values were dealt with.in second phase, decision tree was sued for predicting the potential diabetic patients based on their symptoms. 78% accuracy was produced in this classification. Proposed a Genetic Algorithm based fuzzy model to predict the presence of diabetes disease. In this study, fuzzy model was used as a learning and self-adapting capability [9]. It was used in combination with genetic algorithm to classify the reduced attribute set of diabetes mellitus. It gave an accuracy of 83%. A meta combination of Extreme Learning Machine and Genetic Algorithm was developed for the diagnosis of Pima Indian diabetes [10]. Genetic algorithm was used as an attribute selector while extreme Learning Machine was used for classification, 10-fold cross-validation was used for performance evaluation using metrics like accuracy, specificity and f-score. It generated an accuracy of 89.54%. In [11], degree of occurrence of diabetes is predicted by the use of random forest classifier. Electronic health record of patients are used and analysed to sort the vital symptoms causing diabetes disorders. Then random forest is applied for classification of diabetes. Accuracy obtained was 92%.

A new cascaded learning using Least Square Support Vector Machine (LS-SVM) and Generalized Discriminant Analysis (GDA) was developed for diabetes diagnosis [12]. GDA was used to categorize the patients into healthy and affected with diabetes.LS-SVM was used to efficiently classify the diabetes dataset. The proposed model gave 82.05% accuracy rate with 10-fold cross validation method. An Artificial Neural network-based system and a fuzzy neural network model were proposed by Reference [13] to identify Pima Indian diabetes disease risks effectively. 84.24% was the classification accuracy obtained in this study. A new controller based on fuzzy logic expert domain knowledge system to control blood glucose level was proposed by Reference [14]. it discussed a multiple daily injection regimen (MDIR) for effective treatment of diabetes. It utilized expert domain knowledge by applying fuzzy logic controllers to control the glucose level in blood. It worked in a two loop feedback mechanism. The inner loop regulates the quantity of insulin generation on day today basis while the outer loop supervised the inner loop.

A model based on a stochastic system that presents variability from automated blood glucose level time series is proposed [15]. Here, the interrelationship between the long term associated side effects of diabetes and glucose variability are projected using a stochastic model. The proposed method was later validated and simulated with three different diabetes patient datasets. Researchers have proposed a modified cross-validation method that used an objective function and proposed SVM based optimization techniques which used Particle Swarm Optimization-Support Vector machine (PSO-SVM) and Genetic Algorithm (GA-SVM) [16]. An objective function on the basis of leave-one-out cross-validation was adopted by both of them. Genetic algorithm was used to optimize the parameters of SVM. It was observed that PSO-SVM model was successfully able to handle SVM parameter tuning in a cost effective way. It was successfully applied to a diabetes dataset to classify patients based on relevant symptoms.

In Reference [17], Hasan Temurtas used the Levenberg–Marquardt (LM) method to train the structure of the neural network. The model was used for diabetes disease diagnosis and was compared with several previous works on diabetes diagnosis. It gave an overall prediction accuracy of 82.37%. The integration of fuzzy computing and genetic algorithm to detect presence of diabetes in patients was discussed. Genetic algorithm was used for selecting relevant attributes from the Pima Indian diabetes dataset [18]. Then fuzzy logic was applied to the reduced dataset for proper classification of patients. A mapping of dataset attributes with the use of membership functions based on appropriate measures was carried out. The presented model was evaluated and an accuracy of 87% was produced. A multi-combination of Attribute selection, clustering, Genetic algorithm and decision trees was developed and implemented by Reference [19] for diabetes risk prediction. It combined and built the optimal decision trees on the basis of predefined threshold criteria. It used a lower number of leaf nodes and complexity size was quite less. An accuracy of 83.3% was the output of this hybrid model.

In Reference [20], author has proposed a Rule-based genetic algorithm classifier that optimized the fitness function metric and present a better performance than conventional approaches such as Naive Bayes (NB). Developed a Fuzzy logic-based expert model to predict diabetes based on knowledge of the patient’s history. Fuzzy Logic based Diabetes Diagnosis System (FLDDS) incorporated various parameters for effective diagnosis of diabetic patients. It considered both fuzzy rules generated as well as knowledge of medical experts in predicting diabetes symptoms. The results showed that the performance of the developed fuzzy model increased when the number of parameters and variables are increased. FLDDS model produced an accuracy rate of 87.2% in diabetic dataset [21].

Reference [22] presented an intensified fuzzy expert system for diabetes diagnosis. The system model comprised of fuzzy inference, implication and aggregation module. Knowledge is denoted in fuzzification to transform crisp values into fuzzy values. Fuzzy values is converted back to crisp values by defuzzification. Aim of the proposed fuzzy based model was to enhance the accuracy rate and knowledge quality for diabetes prediction task. This fuzzy system presented effective results with reasonable diabetes data samples and produced a classification accuracy of 88.35%. A classification framework on pattern recognition and rule-based extraction was developed [23]. This method introduced an inverted hierarchical Neuro-fuzzy Binary Space Partitioning (BSP) framework to classify records and extract rule-base from databases. It performed a recursive partitioning of the input feature space and auto-generated its own structure. It permits knowledge extraction with interpretable fuzzy rules. It was evaluated with several datasets and diabetes was one of them. A classification accuracy of 78.26% was produced with diabetes data.

A hybrid combination of different data mining methods for Pima Indian diabetes diagnosis. K-means algorithm was used [24] to validate class label of provided instances. The final classification model using k-fold cross validation was developed using C4.5 algorithms. When evaluated with different existing algorithms, the proposed hybrid prediction model got an accuracy of 92.38%. Reference [25] demonstrated a prediction model by pruning the diabetes dataset. The J48 classifier was used to classify the patients into diabetic and non-diabetic. It aimed to compare the accuracy of prediction of Pima Indian diabetes using multi-layer perceptron with tree based classifiers. J48 classifier gave an optimum accuracy of 89.3% when compared to multi-layer perceptron, which produced 81.9% accuracy. When the attribute ‘number of times pregnant’ was removed from the dataset, the accuracy jumped to 89.7%.

A new variation of MLP called the artificial metaplasty based MLP model (AMMLP) was developed in Reference [26]. The AMMLP model was used to validate a Pima Indian diabetes dataset. The results were compared with other classifiers using the same dataset. It generated an accuracy rate of 89.93%.

An automated diabetes prediction model on a relatively sparse dataset was proposed [27]. The authors propose the Attribute Weighted Support Vector Machines (FW-SVMs) and a Modified Cuckoo search (MCS). Principal component analysis was used to remove irrelevant attributes from the dataset. Then the level of significance of attributes was computed using mutual information method. Later MCS was applied to the dataset to choose the attributes with optimum parameter indices. The reduced optimized set was classified using FW-SVMs. The results presented an accuracy of 93.58%. In Reference [28], researchers have studied the significance of hidden pattern in a variation of the SVM model. A new one class SVM model on the basis of hidden information was derived. The performance was demonstrated with many publicly available datasets and while evaluating on diabetes dataset, it gave a prediction accuracy rate of 87.6%.

Reference [29] presented an effective diagnosis of diabetes using an artificial intelligence approach. In this analysis, a new artificial Bee Colony algorithm was developed to predict the presence of diabetes. In this work, a new blended crossover phase of the genetic algorithm was applied to the chromosomes, which helped in improving the diversity of the ABC algorithm. The results show an accuracy of 91.9%.

An expert system model [30] based on multi-layer fuzzy prototyping to highlight the uncertainty in knowledge was developed. Here a 5-embedded layer of fuzzy system which includes fuzzy group relation, fuzzy group domain, fuzzy group personal domain and fuzzy group ontology layer. It is used to represent uncertain knowledge. It is applied in this study to define and model the knowledge base of diabetes data. The mean accuracy produced after implementing the fuzzy-based expert system was 93.8%. Reference [31] presented an ensemble learning based classification framework for effective prediction and diagnosis of diabetes mellitus. It uses decision stump as the base classifier and the Adaboost module for classification. The presented model was compared with other classifiers like naive Bayes and SVM for validation. The implemented Adaboost classification model showed an accuracy of 84.09%. An extensive comparison analysis among several algorithms was performed [32]. The study focused on the prediction and analysis of gestational diabetes symptoms and relevant Attributes. The data samples consisted of 600 records. The classifiers used were the random tree, decision tree and NB (Naive Bayes). After classification, it was observed that a random tree generated an optimum accuracy of 93%.

The author of Reference [33] presents a new multi-view knowledge gaining approach for the proper diagnosis of Alzheimer’s Disease (AD) using genetics and neuro imaging datasets. At first, a Multi-Layer Multi-View Classification (ML-MVC) method is built to establish the interrelationship between attributes and classes. Then, the Alternating Direction Method of Multipliers (ADMM) was used to solve the minimization issue. The results were validated and it showed good performance with varying datasets. A new survival mechanism was developed [34] where models were trained from historical electronics records. It is helpful in developing potential complications in diabetic patients. A more accurate prediction of symptoms and good ranking of risk factors associated with diabetes are the two vital benefits of this approach. Moreover, a multi task survival framework was presented to analyze the interrelationship between risk factors of the survival approach. At the end, the model was verified with diabetes mellitus diabetes data instances and the performance was recorded. In Reference [35], the authors presented a boosting ensemble classification model for diabetes patients based on their personal as well as medical history data. Random committee classifier was used for the study. A real time diabetes data with 100 records were used and 81% accuracy was the result with 10-fold cross validation method. Reference [36] developed an enhanced non-invasive technique for detection of diabetes disease where a probabilistic classifier with facial key block variables were used the evaluation was performed on a data record with 284 diabetic affected patients and 142 healthy persons. The result indicated that the presented probability model was able to accurately predict the diabetes disorders compared to other seven classification methods used. Researchers discussed a work on diabetes which analyzed the lack of awareness and bad eating habits as the prime factors in developing nations [37]. Six machine learning approaches, which include SVM, regression, neural network, classification tree, naive Bayes and rule classifier, were used for evaluation. Regression produced an accuracy of 78% while neural network gave 77% accuracy.

A traditional technique iridology has been discussed [38] for the treatment of pima Indian diabetes using a machine learning approach. 338 data samples were taken out of which 158 were not affected with diabetes and rest 180 were diabetic patients. Infra-red snaps of eyes were cropped and the desired region of the iris was taken, which correlates to the pancreas location based on an iridology study. The texture and statistical variables were selected from the desired region of interest. Many classifiers were used for the evaluation and it was observed that random forest produced a higher accuracy of 89.63%. In another related work on diabetes, Reference [39] performed a diabetes risk assessment on basis of family background and lifestyle of people. Nine hundred and fifty two data samples were collected from both online as well as offline mode. Diabetes risk was evaluated and demonstrated with six classifiers and random forest model provided the best accuracy of 94.1%. Amelec Viloria [40] asserted that blood sample data was not enough for the effective diagnosis of diabetic patients. This study applied the SVM classifier for treatment of diabetes. The analysis was used on Colombian residents and it produced 99.2% accuracy but it drastically dipped to 65.6% accuracy with people from other ethnic backgrounds. Reference [41] presented five different machine learning models to determine the presence of pima Indian diabetes. Boruta method was used as a wrapper approach for attribute selection which produced an optimized dataset. R language was used for evaluation. It was experimentally shown that all five classifiers gave good performance. Among them, SVM-linear technique gave the highest accuracy of 89% and 88% precision metric. Table 1 highlights a summary of some popular research work studied by authors.

An extensive literature survey was performed primarily based on classification accuracy. Different works used different machine learning approaches for diabetes diagnosis. Common algorithms used in existing research works include decision tree, neural network, SVM, fuzzy logic and ensemble classifiers like random forest. In some of the works, the attribute selection method was also applied. Genetic algorithm was the most common attribute selector used in many research works undertaken.

Among all works analyzed, maximum accuracy was obtained using SVM classifier but its performance also dipped with heterogeneous attributes and data samples [40]. Though in some works, ensemble learning was used, the accuracy rate was still not so high and even if it is good, the execution time delay and other parameters like precision and recall were still not up to standard. Also in many previous works, data preprocessing was not done effectively. Hence there is a need for a more efficient, optimized and productive classification model for diabetes detection and diagnosis. In the next section, the proposed model is discussed in detail.

## 3. Materials and Methods

In this section, the authors aim to clearly present all the requirements and tools needed to obtain the results. Consequently, detailed technical information and pseudo-codes are presented in this section. This research work has been carried using the Waikato Environment for Knowledge Analysis (WEKA) machine learning software tool. This software tool is mainly developed in Java language and is platform-independent. It has a collection of multiple machine learning techniques and algorithms which help to study real-world data analysis problems. This tool requires the dataset to be present in an American Standard Code for Information Interchange (ASCII) text format called ARFF (Attribute-Relation File Format) format. This ARFF constitutes a distribution of various occurrences of attributes and their values in a file. This ARFF file is constructed for data storage in the database, which is then transformed by WEKA and loaded accordingly to perform the experiments. The diabetes dataset used in our study is presented in this ARFF format before performing pre-processing and classification techniques. “weka.classifiers.functions. MultilayerPerceptron” is the WEKA library package used to implement MLP. In this study, the MLP classifier is used to detect the presence of diabetes on the basis of symptoms [42]. It is one of the most reliable, flexible, non-linear and classical categories of neural networks. Two layers of neurons are taken into consideration. The input layer receives the raw data which is moved forward to hidden layers that acts as an abstraction interface. Finally, the output layer is used to predict the class label based on the problem under consideration. The diabetes dataset used is a text based data records available in tabular form. The MLP classifier can learn and model itself in non-linear and complicated problem domains. It can offer generalization ability. Once the model is trained with input data using the MLP classifier, it can establish unseen associations in unseen data instances as well. This is quite helpful in predicting the unseen data. It can also be used in handling larger datasets. The complexity of parameters can be handled by adjusting network complexity and its weight values [43,44]. Since it is non-parametric in nature, error is eliminated in estimating parameters. Apart from these benefits, no limitations are imposed on the overall distribution of the input attributes when using the MLP classifier [45]. Similarly, the “weka.attributeSelection.GeneticSearch” package needs to be loaded to use the Genetic algorithm for the attribute selection task. For effective data analysis and visualization, the JFreeChartis, which is an open-source Java framework, is utilized in our work. The system requirements to implement the results of this research work are quite simple. A 64-bit Windows Operating system with a Quad-core processor and a minimum of 8 GB RAM is the primary requirement while at least Java 1.7 version or higher is required to install WEKA software.

The Pima Indian diabetes dataset is utilized for our research. It is derived initially from the National Institute of Diabetes and Digestive and Kidney Diseases. This dataset constitutes a total of 8 distinct attributes along with 768 instances, as shown in Table 2. All recorded instances belong to female category. Among these attributes, ‘preg’ denotes the pregnancy count which is applicable to women. A high glucose level in the mother can affect the baby during the initial stage of pregnancy. Women with gestational diabetes tend to develop type 2 diabetes in the future. The data samples have missing and redundant values in some attributes column. In attributes ‘preg’ and ‘mass’ many cell values are missing. So an effective and enhanced attribute selection approach is required.

This study includes proposing a progressively productive Attribute selection methodology which is related to the Genetic Search method to identify the presence of diabetes. The authors have defined a probability of 20% and a 2-point crossover. In order to use a probability of 25% after crossover, the 1-bit Mutation is applied. The crossover and mutation rate is predefined for all generations of genetic algorithm. With variation in mutation and crossover rate, a more diverse solution set is obtained thereby making the process more adaptable and dynamic in nature. The variable and dynamic nature of mutation and crossover probability is a major highlight of this proposed Attribute optimization technique.

In our research, there are different abbreviations and parameters used and are part of the proposed model as highlighted in Table 3.

Steps of the proposed algorithm model is here presented. It has four distinct functional units that involve:

- ISS_Gen (FS^initial^, FS^final^) module: Pseudo-code 1 (Table 4) represents the initial binary-encoded solution space.
- Comp_fn(x) module [35]: Pseudo code 2 (Table 5) represents the Enhanced Genetic Search which is a new fitness function.
- Adaptive_CRR-MRR module [47]: Pseudo code 3 (Table 6) represents the dynamic capability of the EAGA algorithm, which is done by changing the CR and MR in every round. [47]
- RS_Mutate module: Pseudo code 4 (Table 7) represents the modified Mutation operation that is based on HOB and LOB.

The pseudo-code of ISS_Gen (FS^initial^, FS^final^) unit, is shown in Table 4, where the raw pool of attributes is used to produce the solution set for the first round. For each attribute, a predefined upper bound is considered. For numerical attributes, the threshold point is the average worth of attribute that is the cause of the disease occurrence. The Attribute value of each character is varied on the basis of threshold value. Observed column values that are less than the mean are defined as 0 and the values higher than the mean are represented as 1, in numerical Attributes. Similarly, for non-numerical Attributes the presence of the disease is labelled as 1 otherwise, it will give 0. Cumulative count of all occurrence of 1′s is done for each column and on the basis of optimum 1′s count for a specific attribute, the relevant attributes are retained. The total 1′s count is done for every column in the table and the attributes with least 1′s count is dropped.

The new fitness function module is generated with the help of compute_f (n) as seen in Table 5. The Fitness function depends on the misprediction rate [47] and total number of zeros in the chromosomes set. The new fitness function is developed based on those two factors and as per the suitability ranking of chromosomes is done. The most priority is given by the least fitness function.

The adapt_CRR-MRR process, as illustrated in Table 6, handles the difference between the rate of crossover and the rate of mutation. At first, it analyzes the initial rate of crossover (CRR) and mutation (MRR). The genetic algorithm prefers an optimum crossover rate with a minimum mutation rate. As the rate of crossover and mutation rate have differed values, for the first-generation rate of crossover and mutation 0.5 were set.

Assume two parent chromosomes undergoes crossover operation. Let f1 and f2 be the fitness value of two parents for the two offspring. The crossover variant (COV) is represented as:

(1) COV=f1−f2.

Average of the crossover variant CMα for a generation with cn crossover rate is [47]:

(2) CMα=1cn∑CM,

where, cn represents the crossover rate for nth round. Consequently, crossover operation for a specific CMα parameter is utilized. So, let mutation variant (MTV) depicts the mutation outcome as:

(3) $$\mathrm{MTV}=f_{\mathrm{new}}-f_{\mathrm{old}},$$

where the fitness function value of the resulting solution is f_new_ and f_old_. The mutation mean (MM) for a round that witness m_n_ transformations are:

(4) MMα=1mn∑MM,

where *n* is the number of bits mutated. These mean values support the probability rate of crossover and mutation to be adapted towards the termination of every iteration. The operators are self-adjusted based on the previous rounds. It proposes that the support of the participants with a higher mean frequency of mean value is more and thereby, the probability improves in the succeeding round and vice versa, which is illustrated below:

**Case 1** [47]:

CM > MM

CRR = CRR + q, MRR = MRR − w

**Case 2** [47]:

CM < MM

CRR = CRR − q, MRR = MRR + w

Here q and w represent the adaptability factors related to CRR and MRR, respectively.

Table 7 highlights the variation of mutation that take place in the last generation as shown in the RS_Mutate unit. It can be applied only for the last round. On the basis of the outcome of crossover activity, the results are correctly analyzed. The fitness factor of chromosomes upturns towards the high order bits (HOB). If solution converges at global optimum if low and if it was more, then the low order bits (LOB) is upturned to make the solutions fine-tune.

Pseudo-code 5 (Table 8) represent EAGA module technique which enhanced and adapts the genetic algorithm technique. The generation of solution space for the first binary coded chromosome is done on basis of ISS_Gen (FS^initial^, FS^final^) module. For each set of solutions, the fitness function is determined by Compute_f (n). The Attributes of fitness function are recorded and stored. To encode the solution set two-point, crossover operation is used and then result of the fitness function after crossover is calculated. On the basis of the computed values of fitness function in the solution set, least priority chromosomes are replaced with better and high priority fitness function solutions. The individual solution is implemented after the crossover. This procedure continues until the predetermined penultimate iterations. According to the RS_Mutate module, a modified procedure is used excluding the last generation. Therefore, after mutation, the last reduced attribute set is the yield that is utilized for characterization. The adaptability characteristic of EAGA is due to variation of CR and MR in each iteration. In light of the estimations of Crossover Mean Variant (CMα) and Mutation Mean Variant (MMα), the mutation and crossover probability is updated in each round. It forms one of the major highlights of the Adaptive_CRR-MRR unit.

Hence, it may be inferred that our developed EAGA model is a combination of two prime constituents which are:

− Chromosomes swapping for optimal fitness value at each iteration.
− Variation in crossover and mutation probability in every iteration.

Based on the first constituent, an Enhanced Genetic Algorithm (E-GA) may be developed which retains the chromosomes with good fitness values as compared to its previous round. It is done by swapping better fitness valued chromosomes of the current round with low fitness valued chromosomes in the last round. Table 9 highlights the E-GA constituent.

Similarly, the pseudo-code 7 (Table 10) highlights the second idea using the Adaptive_CRR-MRR module. A dynamic crossover and mutation adapting scheme can be employed to determine the gain in information regarding the ability of each operation to generate offspring with better fitness values. It is referred to as an Adaptive Genetic Algorithm (A-GA), which varied the crossover and mutation rate in every round based on the performance at its previous round. These variations (A-GA and E-GA) are used for comparative analysis with our proposed EAGA algorithm to demonstrate the algorithm performance.

**Figure 2:**
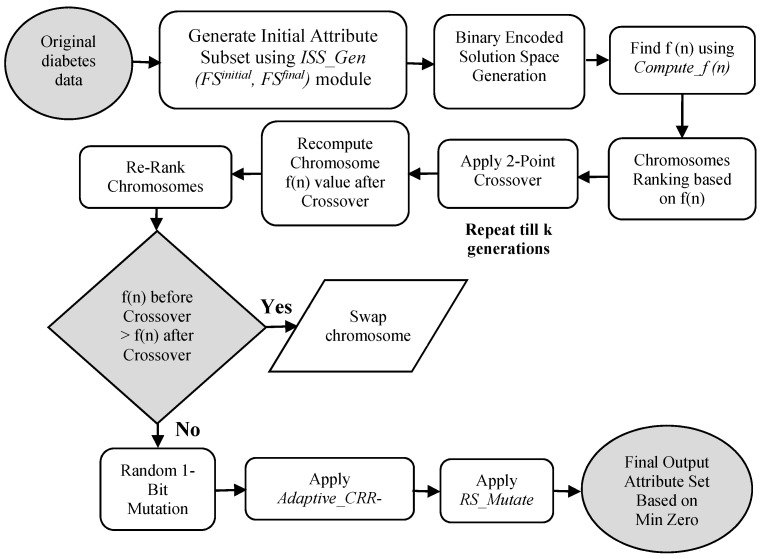
Proposed Optimized Attribute Selection Method.

Figure 2 represents the diagrammatic algorithm of the EG-GA. The input attribute is denoted by the original dataset of Diabetes. The maximum occurrence of 1′s count rule generates a sub-optimal Attributes presented in ISS_Gen (FS^initial^, FS^final^) module. This reduced Attribute set generates the initial chromosome set. Then the fitness function is determined as per the Compute_f (n) module. Subsequently, on the basis of fitness function values, the chromosome ranking is calculated. A 20% probability of chromosomes is performed with 2-point crossover operation. The chromosome’s fitness function after crossover is recalculated while the low priority values are removed from the set of solutions for the successive round. The fitness function is ranked again based on their value. The result set is having a mutation probability of 20% with a 1-bit flip Mutation. In first-generation CR and MR are predefined at 20% while for the resulting generation, these variables are processed by the Adaptive_CRR-MRR unit.

In this unit, the CMα and MMα are resolved and afterward, the CRR and MRR values are balanced as per the needs of the subsequent round. Specified for k iterations, the complete method is repeated. In the final iteration, the Restrict Mutate idea is implemented on the optimized solution set in the RS_Mutate module. For the final attribute set, a total count of the occurrence of 1′s is done. It follows with a maximum of 1′s count policy where the attributes containing a low 1′s count in their corresponding attribute column gets eliminated while remaining attributes are validated, which are further presented as the optimum attribute set.

**Figure 3:**
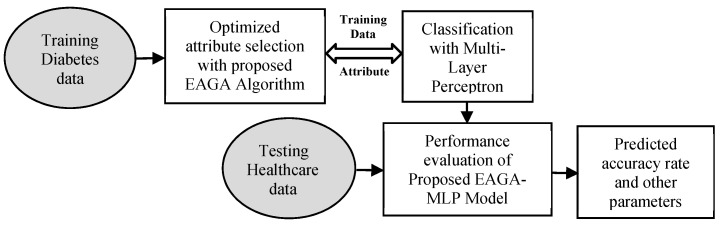
Classification Model based on our Proposed Attribute Selection method (Enhanced and Adaptive-Genetic Algorithm-Multilayer Perceptron (EAGA-MLP)).

Figure 3 represents the proposed novel classification algorithm. The initial raw diabetes data records are the input to the developed optimized attribute selection model. The less relevant attributes are dropped. Simultaneously it collaborates with the neural network, which acts as the classifier and, thereby, a reduced attribute set is the output. The resultant Attribute set is applied with MLP for the classification task. On the basis of this output, the presentation of the categorization is processed utilizing execution metrics and the prediction rate of accuracy is calculated.

## 4. Practical Evaluation

In this section, a practical demonstration of our proposed EAGA algorithm is presented. The sample simulation result of our work is implemented on Diabetes dataset with 8 attributes and 768 instances. The ISS_Gen (FS^initial^, FS^final^) module generates a first reduced attribute set by max 1′s count norm. At first, the mean value is computed and based on that frequency of 1′s for every attribute is found out. Table 11 depicts the mean value calculation for each attribute in diabetes dataset.

The frequency count for each attribute is done in Table 12. It is seen that the frequency counts of 1′s for ‘Pedi’ attribute is only 5, hence it is eliminated. After the removal of the ‘Pedi’ attribute on the basis of lest 1′s count, then reordering of attributes on the basis of 1′s count is performed. In the subsequent step, as shown in Table 13, a solution space is generated, which id dependent on number of attributes in the dataset. After applying ISS_Gen (FS^initial^, FS^final^) module, a solution space is derived from the remaining 7 attributes based on the formula: Solution space count = 2n [where n denotes the cumulative count of attributes in the dataset].

The samples of 10 chromosomes are demonstrated to present the working of the EAGA method. The fitness function for each chromosome is computed and ranked using Compute_f (n) module (Table 14). The less is the fitness function value and more is the priority of that chromosome.

**Figure 4:**
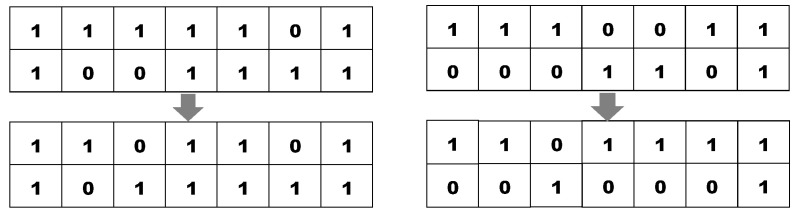
Applying 2-point crossover on sample chromosomes.

The selected chromosomes set are subjected to 2-point crossover step. The crossover probability is fixed at 20%. The crossover operation is shown in Figure 4.

Then following crossover operation, fitness function values are recalculated using the same procedure as presented in Table 15.

On the basis of their fitness function value, chromosomes before and after crossover are compared, as highlighted in Table 16. The lower priority chromosomes are eliminated and better-placed ones are swapped accordingly and placed in the next generation.

It is followed by a random 1-bit mutation with an initial mutation rate of 20% as observed in Table 18. In total, 20% of chromosomes undergo mutation operations, as seen in Table 17.

It is to be noted that the CR and MR for the first generation are predefined (20%). The Adaptive_CRR-MRR module is applied in the subsequent generations for the calculation of CR and MR. Therefore, every round uses a new and dynamic CR and MR. This process continues for k specified generations or until the termination condition is met according to the problem in hand. Then finally RS_Mutate module is used which is a modified version of mutation as highlighted in pseudo-code 4 (Table 5). This ensures that the final output is an optimal attribute set that avoids being trapped in a local optimum (Table 18).

At the end of Restrict Mutate, the frequency counts of 1′s is calculated on every column of the table representing attributes. As can be seen from Table 18, the attribute “mass” has the least count of 1′s (4) and, therefore was eliminated. The final reduced and optimum attribute set after repeating for k generations is denoted in Table 19.

Furthermore, at the end of the simulation, the final ranking of the six attributes in the diabetes dataset is shown in Table 20. This section highlights a sample simulation outcome performed by authors to highlight the working of each step of the proposed EAGA approach. A sample chromosome is taken and each step of the proposed EAGA approach is applied and demonstrated. All the four modules of the presented algorithm are implemented to the sample chromosome and the output is shown in tabular form. The implemented dry run started with the computation of the mean value of each attribute column and is followed by determining the 1′s count for every attribute column. The attribute with the last 1′s count is eliminated from the dataset. The fitness function is calculated as specified in the Compute fn(x) module and the chromosome is ranked according to their priority.

A 2-point crossover is used to the chromosome sample and the fitness function value is recalculated after crossover. On the basis of recalculated fitness function value after the crossover phase, swapping and re-ranking of the chromosome is done, which is subsequently followed by a 1-bit mutation on the chromosome. The process is repeated for the desired iterations. The Restrict Mutate on the resultant chromosome is applied in the last iteration of generation using RS_Mutate module described in Table 5. The final optimized attribute set in the diabetes dataset is the final output after successful completion of all generations. Based on the final attribute set, the ranking is done to eliminate the least relevant attribute from the dataset.

## 5. Result and Analysis

In this research, a recently enhanced and versatile adaptation form of the Genetic Algorithm was created that was referred to EAGA. The newly developed algorithm works on the Pima Indian Diabetes dataset to identify the current status of the patients who have diabetes. Different execution parameters are developed to increase the performance of our proposed model. The algorithm is implemented for 100 generations. In the experimental set up, the network complexity is varied by limiting the values of weight of nodes within a specified range of 0.5. It helps in minimizing the overfitting issue in the MLP classifier that is restricted by varying the complexity of network.

The proposed method has been evaluated using accuracy, latency, precision, recall and F-Score.

The performance of classification is analyzed by the prediction accuracy used as an effective evaluation metric. It denotes the ratio between accurately classified samples and cumulative samples. Equation (5) denotes the accuracy rate.

(5) $$\mathrm{Accuracy}=\frac{\mathrm{Accurately}\_\mathrm{classified}\_\mathrm{diabetic}\_\mathrm{instances}}{\mathrm{Total}\_\mathrm{diabetic}\_\mathrm{instances}}$$

Having a reasonable data classification rate of accuracy is not the main performance parameter. Therefore, other metrics are in a critical stage other than classification accuracy. The performance is the proportion between positive prediction samples and the cumulative data samples of positive prediction. The proportion between the favorable inferences accuracy and the recall ratio of inferences in the class.

Precision and recall are applied for the computation of an optimal technique. Precision represents the actual number of diabetic data samples among all labelled diabetic instances. Equation (6) shows the precision metric.

(6) $$\mathrm{Precision}=\frac{\mathrm{Accurately}\_\mathrm{classified}\_\mathrm{pos}\_\mathrm{diabetic}\_\mathrm{instances}}{\mathrm{Total}\_\mathrm{predicted}\_\mathrm{pos}\_\mathrm{diabetic}\_\mathrm{instances}}$$

Recall denotes the number of correctly predicted diabetic instances among all the diabetic instances present in the dataset. It is highlighted in Equation (7).

(7) $$\mathrm{Recall}=\frac{\mathrm{Accurately}\_\mathrm{classified}\_\mathrm{pos}\_\mathrm{diabetic}\_\mathrm{instances}}{\mathrm{Total}\_\mathrm{diabetic}\_\mathrm{instances}}$$

However, a single metric is required to predict the performance of classification for simplicity purposes. Consequently, F-Score was used to evaluate performance. The harmonic mean of precision and recall is known as F-score. The performance of a classifier is difficult to decide when it might have a better positive predictive value but comparatively lower sensitivity value. In this case, the F-Score is utilized that describes as a balanced average between positive predictive value and sensitivity. An optimal value of F-Score indicates that the algorithm is more efficient. Equation (8) denotes the f-score metric.

(8) $$F-\mathrm{Score}=2*\frac{\mathrm{Precision}*\mathrm{Recall}}{\mathrm{Precision}+\mathrm{Recall}}.$$

The overall latency is processed as the total cumulative time for classification model set up and output prediction period.

(9) Latency=Model_build_time+Disease_prediction_time.

**Figure 5:**
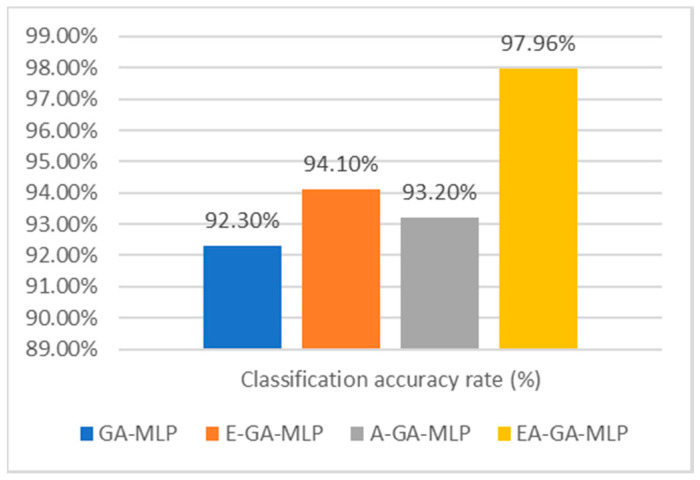
Comparative analysis of accuracy rate of presented algorithm EAGA with its subcomponents.

The performance evaluation of our proposed EAGA algorithm is performed when it is used with the MLP classifier. The sub-constituents of EAGA such as E-GA, A-GA and GA are also tested with MLP classifier to determine its performance. The accuracy rate of the proposed EAGA-MLP is compared with its sub-components. It is observed that classification with EAGA algorithm provides an accuracy of 97.96%, while classification with only the GA has an accuracy rate of 92.3%. The sub-components of EAGA algorithm shows good results and though its accuracy is higher than GA-MLP. However, it is less than the EAGA-MLP model. The classification accuracy is shown in Figure 5.

**Figure 6:**
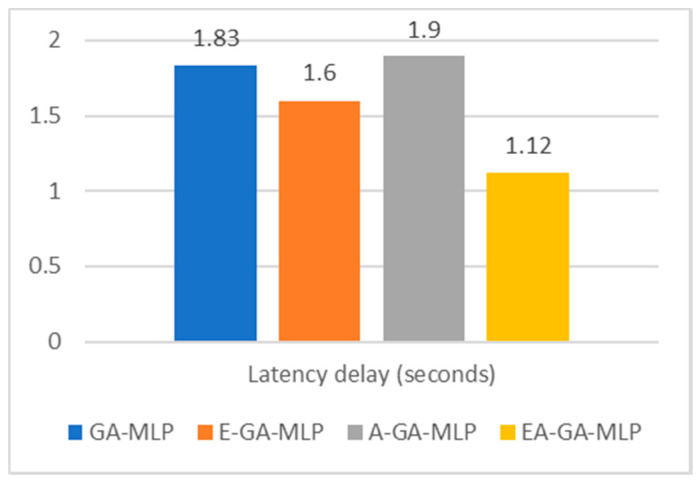
Comparative analysis of latency of presented algorithm EAGA with its subcomponents.

It is also observed from Figure 6 that the EAGA-MLP model is able to perform classification of diabetes patients more proficiently with a minimum time delay of 1.12 sec as compared to its components. The E-GA-MLP takes 1.6 s. However, the A-GA-MLP consumes 1.9 s to execute and generate the classification results.

**Figure 7:**
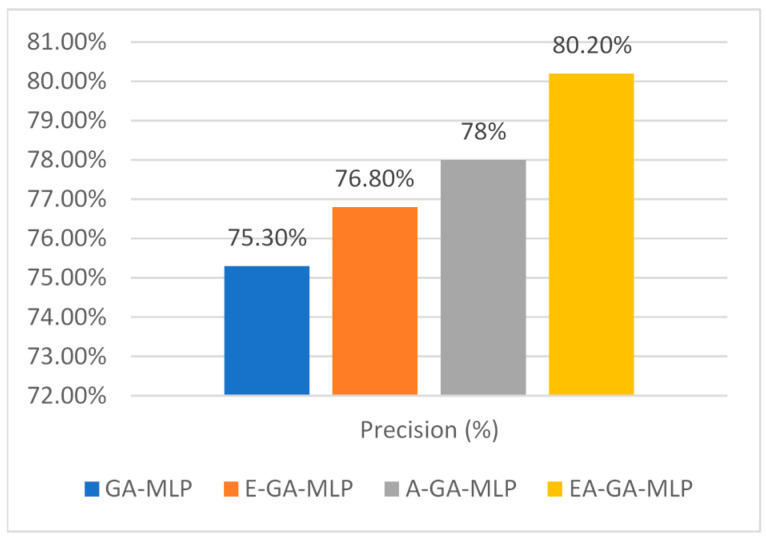
Comparative analysis of Precision of presented Algorithm EAGA with its subcomponents.

The proposed attribute optimization technique also shows a promising result when evaluated with other metrics such as Precision, Recall and F-Score. Figure 7 shows a diagrammatic representation of comparative analysis among the presented works with precision values. The EAGA-MLP model presents a precision value of 80.2%. While the GA-MLP, E-GA-MLP and A-GA-MLP model show 75.3%, 76.8% and 78% value, respectively.

**Figure 8:**
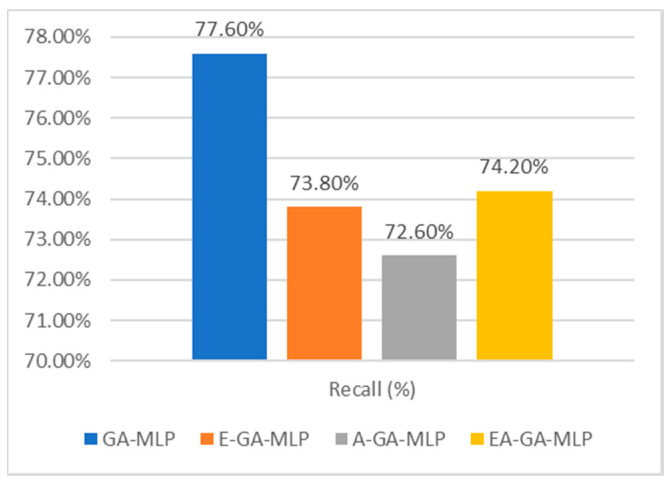
Comparative analysis of Recall of presented Algorithm EA -GA.

The Recall value of our implemented work is recorded at 72.2% after simulation. The GA-MLP model generates the maximum recall value of 77.5%. There is fluctuation observed in recall value of E-GA-MLP and A-GA-MLP models with a minor difference in their recall value. The Recall analysis is depicted in Figure 8.

**Figure 9:**
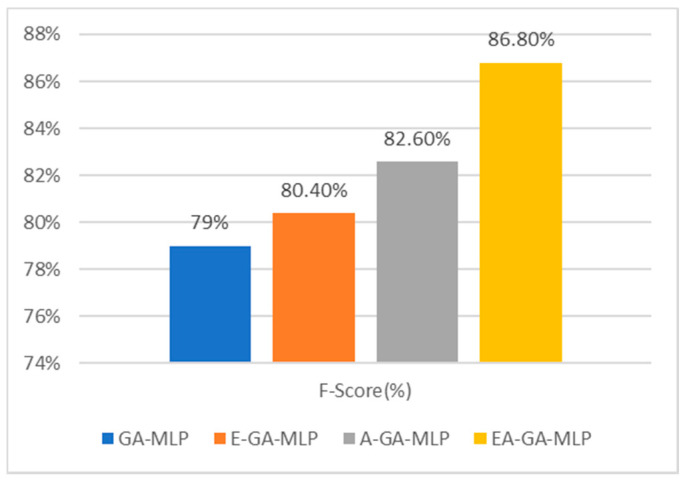
Comparative analysis of F-Score of presented Algorithm EAGA with its subcomponents.

The F-Score metric is a vital parameter for evaluating a classification model. It is the harmonic mean of precision and recall. The recorded estimation of the F-Score value is as high as 86.8% when the MLP classifier worked with the proposed EAGA algorithm. The GA-MLP model shows the least F-Score value of 79%. Also, it is seen that the sub-component A-GA algorithm performs better than the E-GA algorithm when classified with MLP. Figure 9 presents the overall evaluation with the F-Score metric.

**Figure 10:**
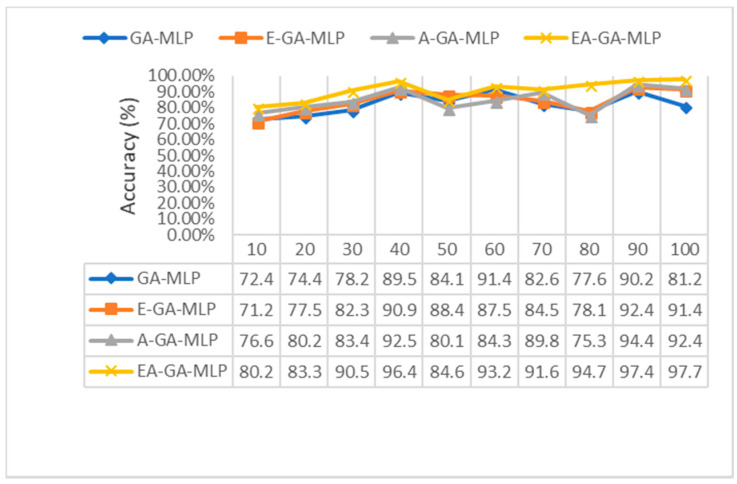
Comparative analysis of Classification Accuracy w.r.t number of Generations.

The EAGA was also tested with the MLP classifier varying the number of iterations and rounds. A comparative analysis of the accuracy rate of EAGA-MLP is carried out with other components (GA--MLP, E-GA-MLP and A-GA-MLP) after every 10 generations. It was noted that after almost every generation, the EAGA-MLP outperforms its constituents. After 10 iteration count, the classification of the EAGA algorithm generates an optimum accuracy rate of 80.22%. Moreover, the GA algorithm shows a reduced accuracy rate of 72.4% after 10 generations. The performance of E-GA and A-GA are also quite average with an accuracy of 71.2% and 76.65%, respectively. However, after 50 iterations, it is observed that the EAGA algorithm performs better than the other constituents producing an accuracy of 84.67% while classification with GA shows an 84.12% accuracy rate. The E-GA constituent generates a maximum of 88.45% classification accuracy, while A-GA constituent shows 80.11% accuracy after 50 generations of simulation. At the end of 100 generations, it is seen that EAGA algorithm produces better classification results with MLP classifier with an accuracy rate of 97.69%. At the same time, its sub-components E-GA and A-GA also show good growth and shows 92.42% and 91.41% accuracy, respectively. The GA produces the least accuracy of 81.23% after completion of 100 generations. The results are summarized in Figure 10.

**Figure 11:**
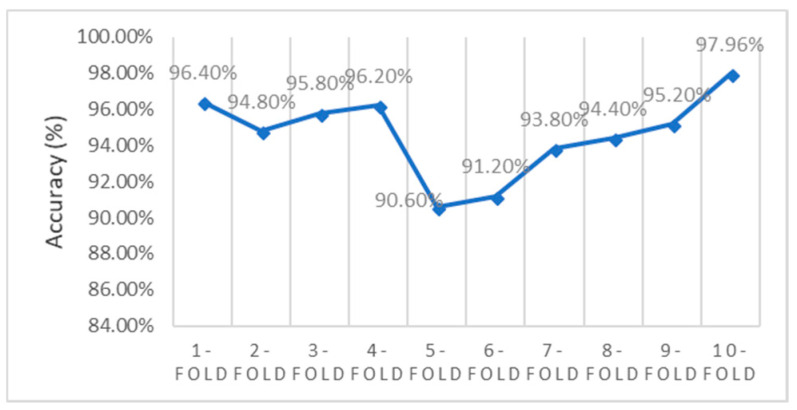
Prediction Accuracy Rate of EAGA method of Cross-Validation technique.

In Figure 11, an experimental evaluation of the prediction accuracy rate of our proposed EAGA algorithm is done concerning the number of folds in the cross-validation method. The training phase was applied with a 10-fold cross-validation. The partition of the informational index was done into 10 sets of equivalent size everywhere 9 sets were prepared as training samples while 1 set is to be used as test data. This process is conducted for ten rounds, thereby the arithmetic mean of prediction accuracy is determined. The proposed EAGA algorithm is classified with the MLP classifier and validated against different folds starting with 1-fold validation to 10-fold validation. Overall it is observed that the accuracy rate is above 96% in every fold of validation. In 1-fold validation, it shows an accuracy rate of 97.7%. Nevertheless, the 3-fold validation method shows a slight dip of accuracy with 96.3% when compared to other folds of the validation method. In the 10th fold of validation, it can be seen that the performance of classification with MLP shoots up to 97.96% accuracy.

A comparison analysis of EAGA concerning other sub constituents was performed with different sizes of data samples. The performance was evaluated with two parameters, which include accuracy and latency to determine the performance of the proposed attribute optimization algorithm. It is observed that the classification accuracy reaches its peak value of 97.96% when the data samples size is 700 while the time taken to predict the disease presence is also a minimum with a value of 1.12 sec compared to other methods with the EAGA method. It is represented in Table 21.

Accuracy analysis of the proposed EAGA-MLP model was done with different literature surveys that are discussed in this study. Several researchers have used different disease datasets in their studies. These studies used different machine learning approaches on variety of healthcare datasets to facilitate treatment of disease disorders and generated reliable accuracy metrics. A comparative analysis is done in Table 22 to show the significant gain in the accuracy rate of EAGA-MLP model. A 93.58% accuracy was obtained by the authors of Reference [27] using FW-SVM approach. 91.3% accuracy was the outcome in Reference [29] where a blended ABC algorithm was used as an attribute evaluator. Similarly, 93.8% accuracy was generated by Reference [30] by implementing a fuzzy expert system on the diabetes dataset. Some other works also generated good accuracy rate as shown in the figure. The EAGA-MLP model shows the optimum accuracy of 97.76% with PIMA Indian diabetes, as compared to other related works noted in the literature survey section.

**Figure 12:**
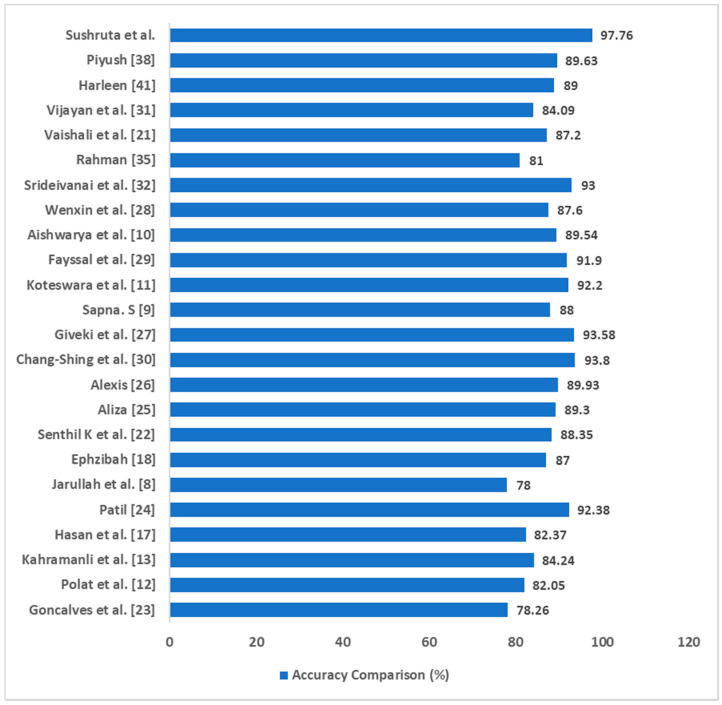
Accuracy comparison of EAGA-MLP model over other literature survey works.

As observed in Figure 12, a 97.6% accuracy is produced by the EAGA-MLP model using the MLP classifier while the fuzzy model [30] gave the second best accuracy of 93.8%. As the accuracy rate is the percentage of correctly classified data samples so a statistical hypothesis test can be applied here with an objective to determine the better model among these two to classify diabetic and non-diabetic patients.

Now from the parameters specified in Table 22, the accuracy of diabetes classification using both models can be computed as follows.

The accuracy rate using the fuzzy model is denoted as: ${\bar{A}}_{1}=y_{1}/m=720/768=93.8$

The accuracy rate using the fuzzy model is denoted as: ${\bar{A}}_{2}=y_{2}/m=750/768=97.6$

Hence the test statistic measure is computed as:

(10) S=A1¯−A2¯2A¯(1−A¯)m,

where $\bar{A}=(y1+y2)/2m$.

The aim is to show that the global accuracy of the EAGA-MLP model (A2) is better than that of fuzzy model (A1) for PIMA Indian diabetes dataset. So accordingly the hypothesis is formulated as:

$$\begin{array}{l} H_{0}:A1=A2\{\mathrm{Null}\;\mathrm{hypothesis}\;\mathrm{denotes}\;\mathrm{both}\;\mathrm{fuzzy}\;\mathrm{and}\;\mathrm{EAGA}-\mathrm{MLP}\;\mathrm{model}\;\mathrm{are}\;\mathrm{equal}\}\\ H_{\beta }:A1<A2\{\mathrm{Alternate}\;\mathrm{hypothesis}\;\mathrm{denoting}\;\mathrm{EAGA}-\mathrm{MLP}\;\mathrm{is}\;\mathrm{better}\;\mathrm{than}\;\mathrm{fuzzy}\;\mathrm{model}\} \end{array}$$

Now the rejection zone is represented as:

$$S<s_{\alpha }\;(\mathrm{if}\;\mathrm{true}\;\mathrm{then}\;\mathrm{reject}\;H_{0}\;\mathrm{and}\;\mathrm{accept}\;H_{\beta }),$$

where $s_{\alpha }$ is derived from normal distribution standard and points to a degree of significance α (predefined value taken as 0.5).

$∴S_{0.5}=1.65$ for 5% significance degree.

It projects that if a norm S < −1.645 is true, it can be inferred with 95% confidence (1−α) that EAGA-MLP model is more accurate than fuzzy model.

Now using Equation (10), the value of test statistic measure is obtained as:

$$S=\frac{93.8-97.6}{\sqrt{2*0.95*0.05/768}}=-307.8.$$

Since, value of S is −307.8 which is much less than −1.645 so it is statistically proved that the alternate hypothesis holds true and the EAGA-MLP model offers a better performance when compared to its next best fuzzy model.

The reliability and generalization of a machine learning model is determined if it performs well with different datasets. The proposed EAGA-MLP model was tested with seven other frequently occurring disease datasets as shown in Table 23. The number and types of attributes differ in these datasets. The instances also vary. It was observed that EAGA-MLP gave a very good accuracy of 95.36% with lesser instances as in lung cancer at the same time it generated 93.76% accuracy with the arrhythmia disease dataset, which had 279 attributes. Hence, irrespective of the number of attributes, instances or types of attributes the proposed EAGA-MLP model gives a steady and high performance with different disease datasets. A mean accuracy of 94.7% was observed. Table 23 highlights the accuracy obtained with the EAGA-MLP model on different disease datasets.

**Figure 13:**
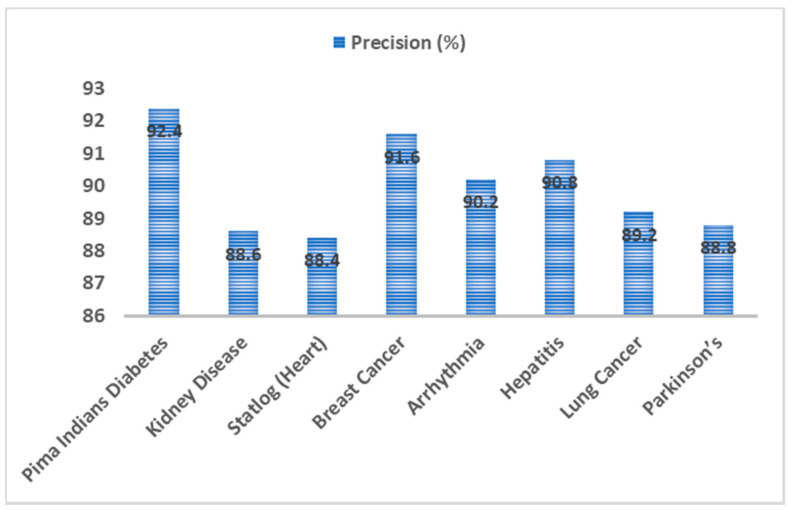
Precision analysis of EAGA-MLP model over different chronic disease datasets.

Figure 13 presents the precision metric comparative study. The precision analysis of EAGA-MLP was also done on these seven disease datasets. An optimum 92.4% was noted with diabetes dataset while the precision value remained almost steady and did not dip significantly with other datasets. The heart disease dataset gave a slightly low precision of 88.4% compared to others. The mean precision obtained after evaluating with different disease datasets is 91%.

**Figure 14:**
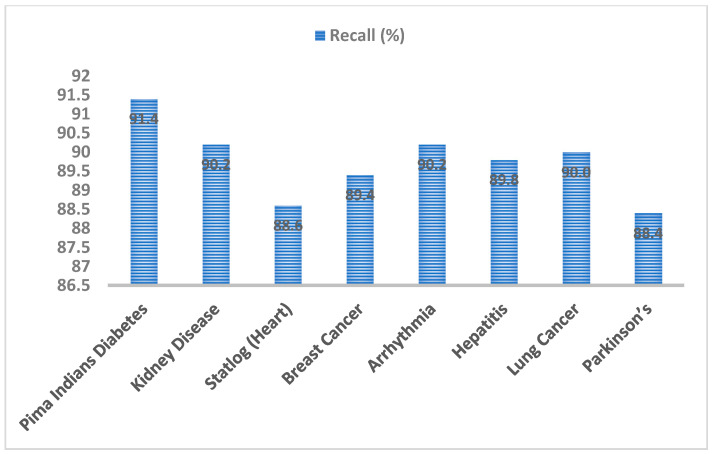
Recall analysis of EAGA-MLP model over different chronic disease datasets.

The recall value represents the proportion of accurately labelled positive values to total actual labels present. The recall measure of the EAGA-MLP model was computed with other disease datasets. Diabetes showed the highest value of 91.4% while the kidney dataset and arrhythmia dataset generated the same value of 90.2%. Lung cancer data gave 90% recall value. Mean recall value computed was 89.8%. Figure 14 highlights the recall metric values obtained with different datasets.

**Figure 15:**
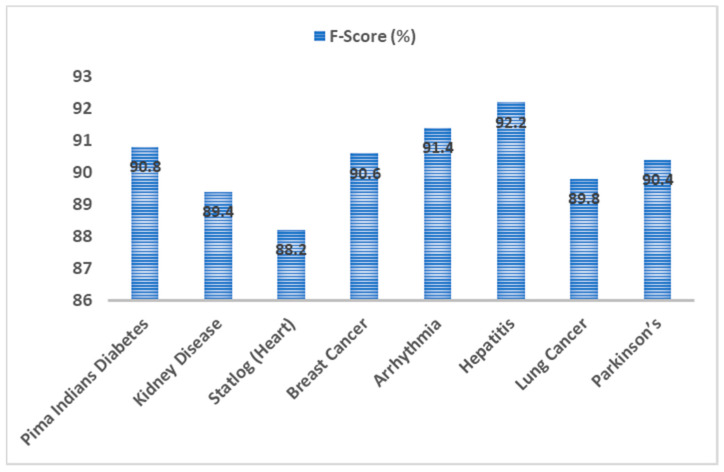
F-Score analysis of EAGA-MLP model over different chronic disease datasets.

The F-Score metric provides a balancing act between precision and recall. It is a more realistic measure to determine the effectiveness of classification. F-score was also used to evaluate the proposed EAGA-MLP technique with other disease datasets taken into consideration. Among the top three f-score value obtained with hepatitis, arrhythmia and diabetes datasets were 92.2%, 91.4% and 90.8%, respectively. The least f-score value with heart disease dataset was 88.2% was. In general, the f-score value was consistent with all the datasets taken in the study. A very high mean f-score of 90.4% was the outcome of evaluation. Figure 15 represents the f-score evaluation.

The proposed EAGA algorithm was effectively implemented in the diabetes dataset and produces an optimized dataset eliminating irrelevant attributes. This reduced attribute set of symptoms was used to classify patients further using MLP classifier to detect the presence of diabetes. The experimental analysis was evaluated against crucial performance metrics such as classification accuracy, latency, precision, recall and F-Score. The EAGA-MLP presents better results when compared with its sub-components (E-GA-MLP, E-GA-MLP or GA-MLP).

The classification accuracy of the EAGA-MLP model was also compared with its components concerning the number of generations. The simulation was performed starting with 10 generations to 100 generations. It was observed that in almost a round of generation, the proposed EAGA-MLP model presents better accuracy than its sub-components.

The cross-validation method was also analyzed and the EAGA-MLP also outperformed its components in terms of accuracy rate.

The size of data samples was also considered for evaluation. With an initial sample of 100 data samples, the data size was enhanced up to as many as 768 data samples. The EAGA-MLP model produced an optimum accuracy rate and minimum latency delay in every size of the diabetic sample.

Statistical analysis was performed to evaluate the performance of EAGA-MLP model. Among literature survey works, fuzzy model gave the best accuracy of 93.8%. Its performance was compared with EAGA-MLP model using hypothesis testing and it was proved that EAGA-MLP model offered better results than fuzzy approach.

Performance analysis of EAGA-MLP model was evaluated with seven other disease datasets to access its performance. Classification accuracy rate of EAGA-MLP was also compared with previous works in the literature survey. The accuracy of the EAGA-MLP model with diabetes, kidney, heart, breast cancer, Arrhythmia, hepatitis, lung cancer, Parkinson’s disease are 97.76%, 94.24%, 95.12%, 94.56%, 93.76%, 94.42%, 95.36% and 92.68%, respectively. Precision observed with diabetes, kidney, heart, breast cancer, Arrhythmia, hepatitis, lung cancer, Parkinson’s disease are 92.4%, 88.6%, 88.4%, 91.6%, 90.2%, 90.8%, 89.2% and 88.8%, respectively. Observed recall value with diabetes, kidney, heart, breast cancer, Arrhythmia, hepatitis, lung cancer, Parkinson’s disease are 91.4%, 90.2%, 88.6%, 89.4%, 90.2%, 89.8%, 90.0% and 88.4% respectively. F-score value noted with diabetes, kidney, heart, breast cancer, Arrhythmia, hepatitis, lung cancer, Parkinson’s disease are 90.8%, 89.4%, 88.2%, 90.6%, 91.4%, 92.2%, 89.8% and 90.4% respectively. The mean value of accuracy, precision, recall and f-score was computed and it was found to be 94.7%, 91%, 89.8% and 90.4% respectively. As it is seen the overall performance was very much consistent with all eight medical datasets.

## 6. Conclusions

This study has presented a novel hybrid model approach of Attribute Optimization called the EAGA technique. This can be called a more enhanced and optimized variation of the Genetic Algorithm which was used with MLP to detect the presence of diabetes in patients. In this work, the Pima Indian Diabetes dataset was used to validate the proposed EAGA approach. The maximum 1′s count rule and a new fitness function evaluation norm were introduced in our proposed algorithm. Chromosome swapping and their ranking on basis of priority are included in the proposed enhanced attribute optimization model. Moreover, 1-bit mutation with 2-point crossover operation was used in this study. A new variation of mutation called Restrict Mutate was applied to the last generation of our EAGA algorithm, which produced promising results.

Multiple evaluation indicators like accuracy rate, latency rate, precision, recall, f-score were analyzed in the study. It is seen that the outcomes are promising. The proposed EAGA-MLP model state an optimum accuracy rate of classification of 97.76% with the least latency delay of only 1.12 sec to execute the classification process. The results show a precision value of 80.2% with EAGA-MLP model. The EAGA-MLP hybrid model presents an F-Score of 86.8%. Moreover, the proposed EAGA is compared with its other constituents and other related existing works of renowned researchers. It is noted that the presented model outperforms similar existing works.

The proposed EAGA-MLP model presents better results when compared with different folds of the cross-validation method, and data sample size. Also the presented model was evaluated and tested with seven other chronic disease datasets like heart disease, breast cancer, kidney disorders among others. The mean accuracy, precision, recall and f-score obtained was 94.7%, 91%, 89.8% and 90.4%, respectively. Thus EAGA-MLP approach can be promising for clinical professionals to accurately determining the presence of Diabetes in patients.

However, this study has limitations. The data sample size and number of generations can be increased to enhance and validate its scalability. Future research will focus on a more progressive, adaptive and execution-oriented approach. The authors aim to update specific characteristics and metrics such as the size of the population. Furthermore, in future, the developed hybrid model can be upgraded and modified for classification of images of different disease disorders such as brain, lungs, thyroid and other real-time image categorization domains.

## Figures and Tables

**Table 1:** Comparative analysis of the accuracy rate of similar existing work.

Authors	Algorithm and Method Used	Accuracy Rate	Year
Goncalves et al. [23]	Hierarchical Neuro-fuzzy BSP system	78.26%	2006
Polat, K., Gunes, S. & Arslan, A. [12]	Generalized Discriminant Analysis (GDA) and Least Square Support Vector Machine (LS-SVM)	82.05%	2008
Kahramanli, H. & Allahverdi, N [13]	Fuzzy neural network (FNN)	84.24%	2008
Hasan Temurtas et al. [17]	LM algorithm and a probabilistic neural network	82.37%	2009
B.M Patil [24]	Hybrid prediction model	92.38%	2010
Jarullah, Al. A. [8]	Decision tree algorithm	78%	2011
E. P. Ephzibah [18]	Fuzzy and genetic algorithms	87%	2011
A.V.Senthil Kumar, M.Kalpana [22]	Intensified Fuzzy Verdict Mechanism	88.35%	2011
Aliza Ahmad [25]	Pruned Decision tree	89.3%	2011
Alexis Marcano-Cedeno [26]	Artificial Metaplasticity based MLP	89.93%	2011
Chang-Shing Lee and Mei-Hui Wang [30]	Fuzzy Expert System	93.8%	2011
Giveki, Davar, et al. [27]	Attribute Weighted Support Vector Machines (FW-SVMs)	93.58%	2012
Sapna. S [9]	Fuzzy and GA	88%	2012
Koteswara Chari et al. [11]	Random forest algorithm	92.2%	2019
Fayssal Beloufa and Chikh [29]	Modified Artificial Bee Colony	91.9%	2013
Aishwarya, S. & Anto, S [10]	Gaussian radial basis function	89.54%	2014
Wenxin Zhu and Ping Zhong [28]	SVM+	87.6%	2014
Srideivanai Nagarajan et al. [32]	Random tree	93%	2014
Rahman Ali [35]	Random committee	81%	2014
Vaishali Jain, Supriya Raheja [21]	Fuzzy Logic-based Diabetes Diagnosis System(FLDDS)	87.2%	2015
Vijayan, V. Veena and C. Anjali. [31]	Adaboost	84.09%	2015
Harleen Kaur [41]	SVM-linear model	89%	2018
Piyush Samant [38]	Iridology technique	89.63%	2018

**Table 2:** Attribute Details of Pima Indian Diabetes [46].

Attribute Name	Labelled Value
Frequency of Pregnancy	Preg
The concentration of Plasma glucose level	Plas
Diastolic blood pressure (mm Hg)	Pres
The thickness of Triceps skin (mm)	Skin
Serum insulin (2-h)	Insu
Body mass index (kg/m^2^)	Mass
Diabetes pedigree function	Pedi
Age (years)	Age
Class label (0 or 1)	Class

**Table 3:** Acronyms discussed in the proposed technique [47].

Name of Metric	Definition
*ISS_Gen*	Initial Solution Set_Generate
*ISS_Gen* (FS^initial^, FS^final^)	Pseudo-code fto generates Initial set of attributes for first round
*Comp_fn (x)*	Pseudo-code for computation of Fitness unit fn (x)
*RS_Mutate*	Pseudo-code for Restrict Mutate unit
*FS^initial^*	Attributes of diabetes dataset at initial stage
*FS^final^*	Attribute set after application of Optimized Genetic Search method
*Attribute_s_*	A threshold value of every attribute to identify if diabetes is present or not.
*Attribute_i_ (worth)*	The merit of individual variable
*Average (Attribute_s_)*	Average worth of every attribute
*OR^max^ (value)*	Optimum Occurrence Rate
*OR^min^ (value)*	A minimum Occurrence Rate
*[1′s count] Attribute_i_*	Number of 1′s in the attribute column
*Attributes ^1′s count (min)^*	Lower indexed 1′s count attribute column
*Attributes^1′s count (max)^*	Higher indexed 1′s count attribute column
*0′s (total)*	Total number of 0′s in a specific solution.
*PR*	Prediction Accuracy
*fn(x)*	Fitness (Evaluation) Function
*1—Prediction accuracy rate*	Misprediction Rate = MPR
ff	fitness factor (0.5)
*z*	No. of 0′s in a specific set of solution
*HOB*	High order bit
*LOB*	Low order bit
*Cross^prob^*	A metric representing the frequency of crossover.
*CRR-MRR*	Crossover Rate-Mutation Rate
*f ‘(n)*	Data structure used in storing pre-crossover fitness unit values [47]
*f “(n)*	Data structure used in storing pre-crossover fitness unit values [47]
*Rank (Soln) _Indiv_*	Ranking order specifying every set of solution based on the fitness function value
*Mutation^prob^*	Metric that indicates the frequency of mutation of a chromosome.
*K*	Number of rounds until the algorithm is executed.
*CM_α_ and MM_α_*	Crossover Mean and Mutation Mean. [47]

**Table 4:** Pseudo-code 1 for ISS_Gen (F^Sinitial^, F^Sfinal^). [47].

**Pseudo Code 1: ISS_Gen (FS^initial^, FS^final^)**
***Step 1:*** *Initialize FS^initial^ = {F1, F2… Fn} for n Attributes*
***Step 2:*** *Determine Threshold for every attribute, Attribute_i_^th^*
***Step 3:*** *Divide the value of the column of every attribute in two parts:*
*Upper limit (> Attribute_i_^th^)*
*Lower limit (< Attribute_i_^th^)*
***Step 4:*** *If attribute == quantitative,*
*Compute Average (Attributei)*
*If Attributei (value) ≥ Mean (Attributei)*
*Attributei (cell) = 1*
*Else*
*Attributei (cell) = 0*
***Step 5:*** *If Attribute! = quantitative,*
*OR^max^ (value) = 1 & OR^min^ (value) = 0*
***Step 6:*** *Compute [1′s count] Attributei (frequency)*
***Step 7:*** *Discard Attributes ^Min. 1′s count^ (Rejection^Prob^ = 6%)*
***Step 8:*** *Calculate FS^final^ = {1- Attributes ^Min. 1′s count^}*

**Table 5:** Pseudo code 2 for Comp_fn(x). [47].

**Pseudo Code 2: Comp_fn(x)**
***Step 1:*** *Develop the solution set for 1st round by ISS_Gen (FS^initial^, FS^final^) module*
***Step 2:*** *Select attributes on basis of class ‘1′ allocation on individual solution*
***Step 3:*** *Determine 0′s (frequency) of every attribute*
***Step 4:*** *Compute PR as:* **Correct predictions/Total predictions**
***Step 5:*** *Find MPR as:* **1-Correct predictions/Total predictions**
***Step 6:*** *Calculate fn(x):* **fn (x) = MPR + qz**
***Step 7:*** *Solutions ranking on basis of fn(x):* **Pre-solution α 1/fn(x)**

**Table 6:** Pseudo-code 3 for Adaptive_CRR-MRR. [47].

**Pseudo-Code 3: Adaptive_CRR-MRR**
***Step 1:* for** *generation ≤ round^max^* **do**
***Step 2:*** Restart a new search set Q^1^;
***Step 3:*** Each set of chromosome in Q; is computed to an evaluation function *fn(x)*
for *|P1| ≤ N* **do**
***Step 4:*** Select 2 parental chromosome from P;
***Step 5:****Crossover happens* in a collection of crossover variant (CV)
***Step 6:****Mutation* happens in a collection of Mutation variant (CV)
***Step 7:*** Add child chromosome to *P_1_*;
**End while**
***Step 8:*** Calculate CMα and MMα and adapt the rate of crossover (CRR) and rate of mutation (MRR);
*Round = Round + 1;*
**End for**

**Table 7:** Pseudo-code 4 for RS_Mutate [47].

**Pseudo-Code 4: RS_Mutate**
***Step 1:*** *Chromosome set post-crossover = input*
***Step 2:*** *Calculate fn(x)*
***Step 3:*** *upturn HOB when fn(x) is low*
*or*
***Step 4:*** *upturn LOB*

**Table 8:** Pseudo-code 5 for EAGA [47].

**Pseudo-Code 5: EAGA**
***Step 1:*** *Start the encoded chromosome set with ISS_Gen (FS^initial^, FS^final^) module*
***Step 2:*** *Accept fn(x) set calculated in Comp_fn(x) module in fn‘(x)*
***Step 3:*** *Apply 2-Point Crossover to the chromosomes on basis of f‘(n) with Adaptive_CRR-MRR unit*
***Step 4:*** *Restore fn(x) determined post-crossover in f ’’n(x)*
***Step 5:*** *fn‘(x) compared to f n‘’(x)*
***Step 6:*** *Swap minimum Rank (Soln) _Indiv_ in fn ‘’(x) with upper Rank (Soln) _Indiv_ in fn ‘(x)*
***Step 7:*** *Iterate till predefined criteria met (k rounds)*
***Step 8:*** *RS_Mutate unit applied to k^th^ phase post step 7*
***Step 9:*** *Calculate reduced attributes ← Attributes^1′s count (max)^*
***Step 10:*** *Result = {Optimal attributes}*

**Table 9:** Pseudo-code 6 for Enhanced Genetic Algorithm (E-GA) [47].

**Pseudo-Code 6: E-GA**
***Step 1:*** *Start with an initial optimized binary coded set of solution*
***Step 2:*** *Save fn(x) calculated in Comp_fn(x) module in fn’(x)*
***Step 3:*** *Chromosomes priority order based on fn(x) value*
***Step 4:*** *Use 2-Point Crossover over chromosome set basis of fn’(x) [20% CRR]*
***Step 5:*** *Compute and reserve fn (x) set determined after Crossover in fn’’(x)*
***Step 6:*** *Evaluate fn’(x) with fn’’(x)*
***Step 7:*** *Reorder the less Rank (Soln) _Indiv_ in f ‘‘(x) to higher Rank (Soln) _Indiv_ in fn ‘(x)*
***Step 8:*** *Use 1-bit Mutation [20% MR] and compute its f (n)*
***Step 9:*** *Reorder chromosomes based on fn (x) value & repeat till criteria satisfied (k rounds)*
***Step 10:*** *Determine Resultant Attribute Set ← Max. 1′s score*
***Step 11:*** *Result = {Optimal attributes}*

**Table 10:** Pseudo-code 7 for Adaptive Genetic Algorithm (A-GA).

**Pseudo-Code 7: A-GA**
***Step 1:*** *Start with the parameters;*
***Step 2:*** *Begin with a random population space P;*
***Step 3:*** *Iteration= 1;*
*while iteration ≤ ^max^ do*
***Step 4:*** *Restart newly generated population P_1_;*
***Step 5:*** *Each set of solution in P; is determined with a fitness value computed as fn(x)*
*while |P1| ≤ M do*
***Step 6:*** *2-parent individuals selected from P;*
***Step 7:*** *Crossover occurs and aggregate Crossover Variant (CV)*
***Step 8:*** *Mutation occurs and aggregate Mutation Variant (CV)*
***Step 9:*** *Push the child solutions to P1;*
***Step 10:*** *Find CMα and MMα and adjust Crossover Rate (CRR) and Mutation Rate (MRR)*
*Iteration = Iteration + 1;*
*stop*

**Table 11:** Calculation of Mean value for each column (attribute).

	Preg	Plas	Pres	Skin	Insu	Mass	Pedi	Age
	5	166	72	19	175	25.8	0.587	51
	5	97	60	23	0	28.2	0.423	22
	7	114	66	0	0	32.8	0.258	42
	1	89	76	34	37	32.2	0.192	23
	8	183	64	0	0	23.3	0.672	32
	7	160	54	32	175	30.5	0.588	39
	4	146	85	27	100	28.9	0.189	27
	13	126	90	0	0	43.4	0.583	42
	2	197	70	45	543	30.5	0.158	53
	3	83	58	31	18	34.3	0.336	25
	2	141	58	34	128	25.4	0.699	24
	15	136	70	32	110	37.1	0.153	43
	2	110	74	29	125	32.4	0.698	27
	3	120	70	30	135	42.9	0.452	30
	4	173	70	14	168	29.7	0.361	35
Mean	5	136	69	23	114	32.1	0.425	34.4

**Table 12:** Calculation of 1′s count for each column (attribute).

	Preg	Plas	Pres	Skin	Insu	Mass	Pedi	Age
	1	1	1	0	0	0	1	1
	1	0	0	1	0	0	0	0
	1	0	0	0	0	1	0	1
	0	0	1	1	0	0	0	0
	1	1	0	0	0	0	1	0
	1	1	0	1	1	0	1	1
	0	1	1	1	0	0	0	0
	1	0	1	0	0	1	1	1
	0	1	1	1	1	0	0	1
	0	0	0	1	0	1	0	0
	0	1	0	1	1	0	1	0
	1	1	1	1	0	1	0	1
	0	0	1	1	1	1	1	1
	0	0	1	1	1	1	0	0
	0	1	1	0	1	0	0	0
Mean	5	136	69	23	114	32.1	0.425	34.2
1′s count	7	8	9	10	6	7	5	7

**Table 13:** Sample Chromosomes are taken at random.

Preg	Plas	Pres	Skin	Insu	Mass	Age
1	0	1	1	0	1	0
1	1	1	1	1	0	1
0	0	0	1	1	0	1
1	0	0	1	0	0	1
1	0	0	1	1	1	1
1	1	1	0	0	1	1
0	0	1	0	1	1	1
1	1	1	1	0	1	0
0	1	1	0	1	0	0
0	1	0	1	1	0	0

**Table 14:** Priority-based chromosome ranking based on Fitness Function.

Preg	Plas	Pres	Skin	Insu	Mass	Age	f (n)
1	1	1	1	1	0	1	18%
1	0	0	1	1	1	1	21%
1	1	1	0	0	1	1	29%
0	0	0	1	1	0	1	33%
1	1	1	1	0	1	0	34%
1	0	0	1	0	0	1	37%
1	1	1	1	0	0	1	38%
0	0	1	0	1	1	1	41%
0	1	0	1	1	0	0	42%
0	1	1	0	1	0	0	48%

**Table 15:** Recalculation of Fitness function after Crossover on the chromosome set.

Preg	Plas	Pres	Skin	Insu	Mass	Age	f (n)
1	1	0	1	1	0	1	25%
1	0	1	1	1	1	1	19%
1	1	0	1	1	1	1	17%
0	0	1	0	0	0	1	44%
1	1	0	1	0	1	0	35%
1	0	1	1	0	0	1	20%
1	1	1	0	1	0	1	21%
0	0	1	1	0	1	1	26%
0	1	0	1	1	0	0	42%
0	1	1	0	1	0	0	48%

**Table 16:** Swapping and Ranking of chromosome set based on Recalculated Fitness function after Crossover.

Preg	Plas	Pres	Skin	Insu	Mass	Age	f (n)
1	1	0	1	1	1	1	17%
1	1	1	1	1	0	1	18%
1	0	1	1	1	1	1	19%
1	0	1	1	0	0	1	20%
1	1	1	0	1	0	1	21%
1	1	0	1	1	0	1	25%
0	0	1	1	0	1	1	26%
1	1	1	0	0	1	1	29%
1	1	0	1	0	1	0	35%
0	1	0	1	1	0	0	42%

**Table 17:** 1-bit Mutation of the chromosome set.

Preg	Plas	Pres	Skin	Insu	Mass	Age
1	1	0	1	1	1	1
0	1	1	1	1	0	1
1	0	1	1	1	1	1
1	0	1	1	0	0	1
1	1	1	0	1	0	1
1	1	0	1	1	0	1
0	0	1	1	0	0	1
1	1	1	0	0	1	1
1	1	0	1	0	1	0
0	1	0	1	1	0	0

**Table 18:** Applying Restrict Mutate on the Chromosome set in the last generation.

Preg	Plas	Pres	Skin	Insu	Mass	Age
1	1	0	1	1	1	1
0	1	1	1	1	0	1
1	0	0	1	1	0	1
0	0	1	1	0	0	1
1	1	1	0	1	0	0
1	1	0	1	1	1	1
1	0	1	1	0	0	0
1	1	1	0	0	1	1
1	1	1	1	0	1	0
0	1	0	1	1	0	0

**Table 19:** Final Optimal and Enhanced Attribute set after k generations.

	Preg	Plas	Pres	Skin	Insu	Mass	Age
	1	1	0	1	1	1	1
	0	1	1	1	1	0	1
	1	0	0	1	1	0	1
	0	0	1	0	1	0	1
	1	1	1	1	0	0	0
	1	1	0	1	1	1	1
	1	0	1	0	1	0	0
	1	1	1	0	0	1	1
	1	1	1	0	1	1	0
	0	1	0	1	1	0	0
1′s count	7	7	6	6	8	4	6

**Table 20:** Final Ranking of attributes after the termination of EAGA.

Preg	Plas	Pres	Skin	Insu	Age	
7	7	6	6	8	6	1′s count
3	2	4	5	1	6	Ranking of Attributes

**Table 21:** Comparative analysis of classification accuracy of EAGA with GA, E-GA and A-GA.

Data Samples Size	Performance	GA-MLP	E-GA-MLP	A-GA-MLP	EAGA-MLP
100	Accuracy (%)	91.46	92.76	93.02	94.02
	Latency (s)	0.05	0.03	1.06	0.06
200	Accuracy (%)	94.47	94.43	94.87	95.17
	Latency (s)	0.88	0.78	0.82	0.8
300	Accuracy (%)	90.98	91.98	92.32	94.32
	Latency (s)	0.99	0.93	0.95	0.75
400	Accuracy (%)	87.26	89.56	90.31	94.51
	Latency (s)	1.53	1.03	0.97	0.9
500	Accuracy (%)	89.78	91.78	89.67	91.67
	Latency (s)	1.73	1.23	1.76	1.04
600	Accuracy (%)	86.33	88.65	89.22	95.22
	Latency (s)	2.07	1.77	1.89	1.89
700	Accuracy (%)	92.24	94.14	93.29	97.96
	Latency (s)	1.86	1.56	1.92	1.12

**Table 22:** Parameters for Statistical hypothesis analysis.

Variable	Description
m	Number of data samples in diabetes dataset
y_1_	Number of correctly classified samples using fuzzy model
y_2_	Number of correctly classified samples using EAGA-MLP model
${\bar{A}}_{1}$	Accuracy obtained using fuzzy model
${\bar{A}}_{2}$	Accuracy obtained using EAGA-MLP model
S	Test statistic measure

**Table 23:** Performance analysis of EAGA-MLP model with different chronic disease datasets.

Disease Dataset	Attributes Types	Instances	Attributes	Accuracy (%)
Pima Indians Diabetes	Integer, Real	768	8	97.76
Kidney Disease	Real	400	25	94.24
Statlog (Heart)	Categorical, Real	270	13	95.12
Breast Cancer	Real	569	32	94.56
Arrhythmia	Categorical, Integer	452	279	93.76
Hepatitis	Categorical, Integer	155	19	94.42
Lung Cancer	Integer	32	56	95.36
Parkinson’s	Real	197	23	92.68
